# Vascular Endothelial Growth Factor and Monocyte Chemoattractant Protein-1 Levels Unaltered in Symptomatic Atherosclerotic Carotid Plaque Patients from North India

**DOI:** 10.3389/fneur.2013.00027

**Published:** 2013-04-02

**Authors:** Dheeraj Khurana, Deepali Mathur, Sudesh Prabhakar, Keshav Thakur, Akshay Anand

**Affiliations:** ^1^Department of Neurology, Post Graduate Institute of Medical Education and ResearchChandigarh, India

**Keywords:** carotid atherosclerotic plaque, vascular endothelial growth factor, serum protein levels, monocyte chemoattractant protein, enzyme-linked immunosorbent assay

## Abstract

We aimed to identify the role of vascular endothelial growth factor (VEGF) and monocyte chemoattractant protein (MCP-1) as a serum biomarker of symptomatic carotid atherosclerotic plaque in North Indian population. Individuals with symptomatic carotid atherosclerotic plaque have high risk of ischemic stroke. Previous studies from western countries have shown an association between VEGF and MCP-1 levels and the incidence of ischemic stroke. In this study, venous blood from 110 human subjects was collected, 57 blood samples of which were obtained from patients with carotid plaques, 38 neurological controls without carotid plaques, and another 15 healthy controls who had no history of serious illness. Serum VEGF and MCP-1 levels were measured using commercially available enzyme-linked immunosorbent assay. We also correlated the data clinically and carried out risk factor analysis based on the detailed questionnaire obtained from each patient. For risk factor analysis, a total of 70 symptomatic carotid plaque cases and equal number of age and sex matched healthy controls were analyzed. We found that serum VEGF levels in carotid plaque patients did not show any significant change when compared to either of the controls. Similarly, there was no significant upregulation of MCP-1 in the serum of these patients. The risk factor analysis revealed that hypertension, diabetes, and physical inactivity were the main correlates of carotid atherosclerosis (*p* < 0.05). Prevalence of patients was higher residing in urban areas as compared to rural region. We also found that patients coming from mountain region were relatively less vulnerable to cerebral atherosclerosis as compared to the ones residing at non mountain region. On the contrary, smoking, obesity, dyslipidemia, alcohol consumption, and tobacco chewing were not observed as the determinants of carotid atherosclerosis risk in North India (*p* > 0.05*)*. We conclude that the pathogenesis of carotid plaques may progress independent of these inflammatory molecules. In parallel, risk factor analysis indicates hypertension, diabetes, and sedentary lifestyle as the most significant risk factors of ischemic stroke identified in North India. This could be helpful in early identification of subjects at risk for stroke and devising health care strategies.

## Introduction

Stroke continues to be the principal contributor of functional impairment and disability in adults and is the second leading cause of death worldwide (Feigin, 2005). It is characterized by a sudden reduction of blood flow in an area of the brain resulting in neurological deficits. Ischemia can produce a transient ischemic attack (TIA) (Kiely et al., 1993) which was defined as acute onset of focal neurological deficit lasting less than 24 h. Deposition of atherosclerotic lesions/plaque in carotid arteries may produce a high risk of ischemic stroke (Aydiner et al., 2007). Therefore, the identification of molecular biomarkers in serum of patients presenting with carotid plaque would assist in the early detection of patients at risk for the ischemic stroke.

Vascular endothelial growth factor (VEGF) has been strongly implicated in brain ischemia (Cobbs et al., 1998). It plays a variety of roles in the disease process, such as forming new and porous blood vessels through a process known as angiogenesis, which stimulates endothelial cells to proliferate and migrate to areas of the brain affected by ischemia (Ferrara et al., 1991; Dvorak et al., 1995; Nagy et al., 2002; Hoeben et al., 2004). In addition to its known role as an angiogenic growth factor, VEGF confers neuroprotection by reducing the neurological damage that occurs after ischemic insult (Yang et al., 2012). VEGF and its receptor VEGFR1/soluble form of full-length transmembrane receptor (sflt-1) are expressed at significantly higher levels in rat neurons after occlusion of the middle cerebral artery (Lennmyr et al., 1998). Analysis of human post-mortem brain tissue after an ischemic stroke has shown that different isoforms of VEGF, including VEGF165 and VEGF189, as well as their soluble receptors, are expressed at higher levels than in samples from patients without ischemic stroke (Krupinski et al., 1999). Because VEGF plays a crucial role in physiological and pathophysiological angiogenesis, measurement of VEGF in serum is of diagnostic and prognostic value as a marker for atherothrombotic disease. Moreover, inflammatory cytokines that are induced by VEGF, such as monocyte chemoattractant protein (MCP-1) have been previously shown to be involved in the pathogenesis and progression of carotid atherosclerosis (Yamada et al., 2003). Therefore, to further elucidate the role of VEGF and MCP-1 as potential biomarkers in ischemic stroke, we sought to estimate their levels in the sera of stroke patients presenting with carotid atherosclerotic plaques in the North Indian population.

## Materials and Methods

The study was initiated after getting approval from the ethics committee of the Institute. The study was conducted in the Doppler laboratory, Department of Neurology, Post Graduate Institute of Medical Education and Research (PGIMER), Chandigarh, India.

Following were the Inclusion criteria for enrollment:

1. Patients of ischemic stroke/TIA > 15 years of age
2. Stroke in the anterior circulation confirmed by neuroimaging-Cranial CT or MRI scan
3. Presence of extracranial atherosclerotic disease on cervical duplex ultrasound
4. Fully informed consent available

The Exclusion criteria were:

1. Patients of hemorrhagic stroke or venous strokes
2. Patients with cardioembolic strokes or family history of thrombotic predisposition
3. Patients with short neck, bony abnormality precluding a cervical duplex study
4. Patients with a high cervical bifurcation on duplex ultrasound
5. Pregnancy

### Subjects for VEGF and MCP-1 estimation

We defined symptomatic carotid atherosclerotic plaque as aggregation of plasma lipids (especially cholesterol), cells (smooth muscle cells and monocytes/macrophages), and connective tissue matrix (proteoglycans) in carotid artery as detected by duplex ultrasound (Garcia and Khang-Loon, 1996). Symptomatic patients with age above 15 years; TIA; left or right hemiparesis underwent duplex ultrasound with carotid atherosclerotic plaque. Detected in anterior circulation were included in the study.

Patients who had symptoms of TIA but were not positive for carotid plaque as reported by Doppler ultrasound constituted the neurological controls. All volunteer asymptomatic family members and individuals who accompanied the patients with no history of serious illness constituted the healthy control group. Healthy controls were not subjected to Doppler examination. However, after informed consent, their blood samples were collected and further processed in the neuroscience research lab. The blood samples of all the patients as well as controls were collected randomly without considering any time limit. It should be noted that the patients who came for follow up a few months after they encountered stroke were also included in the study. Table 1 shows the study population for protein estimation and the duration of disease for patients and neurological controls.

**Table 1:** **Study Population for VEGF and MCP-1 estimation**.

	Sample size (*n*)	Number of symptomatic patients and controls related to duration of disease			
		1–3 weeks	1–6 months	7–12 months	More than 12 months
Stroke (with carotid plaque)	57	7	30	5	8
Neurological controls (without carotid plaque)	38	6	19	4	9
Healthy controls	15				

The severity of carotid atherosclerosis was graded according to the stenosis percentage. (1) Mild: with intima media thickness (IMT) >0.08 cm in CCA (common carotid artery) (2) Moderate: stenosis <50% in ICA (internal carotid artery) (3) Severe: stenosis >50% in ICA (internal carotid artery).

#### VEGF and MCP-1 estimation

Vascular endothelial growth factor and MCP-1 protein levels secreted in the serum of ischemic stroke patients and controls were quantified by enzyme-linked immune assay (Quantikine kits obtained from R&D Systems). All samples were analyzed in duplicate and subsequently used in all further statistical analysis. The assay sensitivity was 5.0 pg/ml for VEGF and 9.0 pg/ml for MCP-1.

#### Total protein and bovine serum albumin estimation

Total protein and albumin were estimated using Biorad protein assay kit. Five dilutions of a protein standard (representing protein solution to be tested) were prepared. Protein solutions were assayed in duplicate at linear range of 8.0 mg/ml to 80 mg/ml approximately. Standard and sample solution of 160 ml each were dispensed into different microtiter plate wells followed by addition of 40 ml of dye reagent concentrate. Multi channel pipet (to dispense the reagent) was used to mix sample and reagent thoroughly followed by incubation for 5 min at room temperature. Then absorbance was measured using microplate enzyme-linked immunosorbent assay (ELISA) reader at 595 nm and samples were analyzed for total protein. Levels of VEGF and MCP-1 were normalized to total protein and further subjected to statistical analysis.

### Risk factor analysis

The patients with carotid atherosclerotic disease and equal number of age and sex matched healthy controls were analyzed. All the IS patients were interviewed after the duplex ultrasound and the clinical and socio-demographic details including hypertension, diabetes, and lipid profile were tabulated in a pre-validated questionnaire. Socio-demographic details such as body mass index (BMI), alcohol consumption, tobacco chewing, smoking, physical inactivity, geographic location (rural/urban), topographic region (mountaineer/plain) was also included. Similar details were collected from controls. Proper informed consent was taken from all the participants. Those who were unable to give their consent were recruited on the basis of an accompanying person’s signed agreement. Illiterate people who were not capable to read the agreement were explained the content of the form verbally and were asked to place their thumbprints on the form.

### Measures

Age and gender of the patients and controls was recorded in the clinical *pro forma*. Most of the variables are self explanatory or otherwise stated.

### Clinical parameters

#### Hypertension

Patients were categorized as hypertensive if the blood pressure was more than 140/90 mmHg or there was a history of receiving anti-hypertensive medications.

#### Diabetes mellitus

Patients were interviewed whether they were diabetic or non-diabetic. They were confirmed diabetic if their fasting plasma glucose was more than 126 mg/dl as per their reports or if they were receiving any anti-diabetic medications.

#### Lipid profile

Levels of total cholesterol, high-density lipoprotein (HDL), low-density lipoprotein (LDL), and triglycerides were noted from patients. Patients who did not have lipid test reports were asked to undergo the test and report the levels.

### Socio-demographic parameters

#### Body mass index

Patients whose BMI was in the range 25–29.9 were considered overweight and whose BMI fell between 30 and 34.9 were obese.

#### Smoking

Smoking was classified into two categories: current smokers and non-smokers. Current smokers represented as reported when interviewed. Former smokers included those who smoked for a period of 10 years before stroke onset. Non-smokers were defined as those who had never smoked in their lifetime and the ones who had quit for more than 10 years before the onset of disease.

#### Physical inactivity

On the basis of low (only walking), moderate, or vigorous exercise, patients were classified as physically active and non-active. Low (only walking) activity was under physically inactive category while moderate and vigorous activity was grouped under physically active category.

#### Alcohol consumption

Patients were categorized into alcohol consumers and non-alcohol consumers on the basis of alcohol intake. Amount of alcohol was also recorded in the questionnaire from alcohol consumers.

### Statistical analysis

For VEGF and MCP-1 estimation, Mann–Whitney *U* test was used and *p* < 0.05 was considered statistically significant. Chi-square test (Pearson Uncorrected) was used as a test of significance for socio-demograhic analysis and *p*-value less than 0.05 was considered as significant. Whenever the values in any of the cells of the contingency table were below 10 Fisher’s exact test was applied.

## Results

The study population comprised of 110 subjects of whom 57 symptomatic patients with carotid plaque, 38 symptomatic neurological controls without carotid plaque, and asymptomatic 15 healthy controls were enrolled for VEGF and MCP-1 estimation. Baseline characteristics of the study population for risk factor analysis are described in Table 2. Prevalence of IS in North India was found more in men than women (71.4% in men). The disease is more commonly seen in elderly people (51.4%). Our results revealed that there was no significant upregulation of VEGF in carotid plaque cases as compared to controls. We also studied MCP-1 levels but here also we did not observe any significant upregulation.

**Table 2:** **Baseline characteristics of study population for clinical/socio-demographic analysis**.

Number of carotid plaque patients	70
Men	50 (71.4%)
Mean age (years)	59.3 ± 12.2 years
Range (years)	
30–39	2.9%
40–49	17.6%
50–59	27.9%
60 and above	51.4%

### Serum VEGF levels in carotid plaque patients

**Figure 1:**
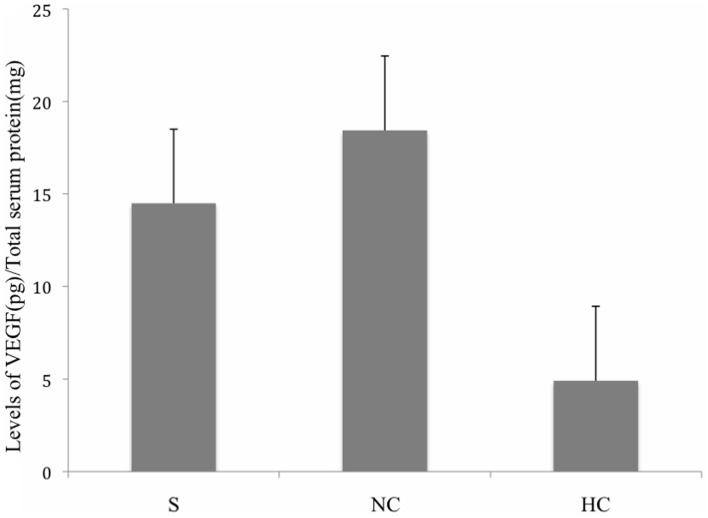
**Levels of VEGF in serum of stroke patients, healthy controls, and neurological controls**. Group means were plotted ±SE. No significant difference was observed among the given conditions (*p* > 0.05). Data was analyzed by Mann–Whitney Test. Levels of VEGF were normalized to total serum protein. (S, Stroke Patients; NC, Neurological Controls; HC, Healthy Controls).

The mean of the VEGF concentration in serum of carotid plaque patients versus healthy controls was 9.7 ± 0.798 pg/ml while the mean VEGF concentration in serum of carotid plaque patients versus neurological controls was 16.468 ± 1.48 pg/ml. VEGF levels were not found to be significantly elevated in patients as compared to either of the controls (*p* > 0.05) (Figure 1).

### Serum MCP-1 levels in carotid plaque patients

**Figure 2:**
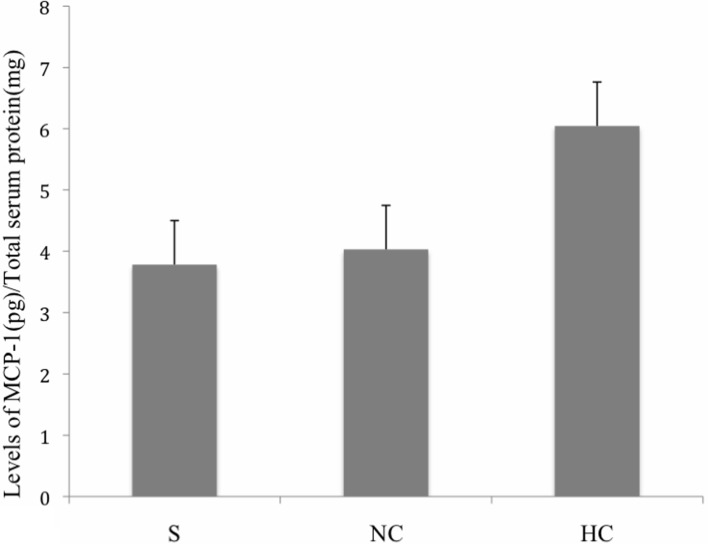
**Levels of MCP-1 in serum of stroke patients, neurological control, and healthy control subjects**. Group means were plotted ±SE. No significant difference was observed among the given conditions (*p* > 0.05). Data was analyzed by Mann–Whitney Test. Levels of VEGF were normalized to total serum protein. (S, Stroke Patients; NC, Neurological Controls; HC, Healthy Controls).

The mean of the MCP-1 concentration in serum of carotid plaque patients versus healthy controls was 4.913 ± 0.22 pg/ml while the mean MCP-1 concentration in serum of carotid plaque patients versus neurological controls was 3.904 pg/ml. We did not observe any significant alteration in MCP-1 levels when compared to either of the controls (*p* > 0.05) (Figure 2).

### Geographical distribution, demography, and risk factor analysis

Clinical details and socio-demographic characteristics of carotid plaque patients in North India are described in Table 3. The risk factor analysis revealed that hypertension, diabetes, and physical inactivity were the main correlates of carotid atherosclerosis (*p* < 0.05). Prevalence of patients was higher residing in urban areas as compared to rural region. We also found that patients coming from mountaineer region were relatively less vulnerable to cerebral atherosclerosis as compared to the ones residing in non-mountainous region. On the contrary, smoking, obesity, dyslipidemia, alcohol consumption, and tobacco chewing were not observed as the determinants of carotid atherosclerosis risk in North India (*p* > 0.05*)*. Table 4 illustrates the odd ratios, relative risk at 95% confidence interval and the *p*-values of clinical and socio-demographic parameters.

**Table 3:** **Clinical details and socio-demographic characteristics of ischemic stroke patients in North India**.

Clinical details	Prevalence (%)	Socio-demographic characteristics	Prevalence (%)
Hypertension	72.7	Smoking	20.5
Diabetes	28.7	Alcohol consumption	29.4
Total cholesterol		Physical inactivity	82.08
250–390 mg/dl (high)	5	Tobacco consumption	6
<250	97	Obesity	6
HDL cholesterol		Fish consumption	33.8
<40 mg/dl (high risk)	38.8	Non-vegetarian	56
>40 mg/dl	61.1	BMI; overweight subjects	28
Triglycerides		Topography (plain region)	88.05
>225 mg/dl (high risk)	8.5	Geographic location (urban)	70
<225 mg/dl	88.5		
LDL cholesterol			
>160 mg/dl (high risk)	4.8		
<160 mg/dl (protective)	95		

*LDL, low-density lipoprotein; HDL, high-density lipoprotein; BMI, body mass index*.

**Table 4:** **Risk factors of IS with odd ratios, relative risks at 95% CI and *p*-values; *p*-values < 0.05 significant**.

Variables	Odds ratio (OR) 95% confidence interval	Relative risks (RR) 95% confidence interval	*p*-value
*Hypertension	12.06 (4.7–31.5)	3.06 *(*2.04–4.46*)*	<0.001
*Diabetes^#^	4.68 (1.50–15.5)	1.73 (1.19–2.13)	0.003
Alcohol consumption	1.26 (0.57–2.79)	1.11 (0.76–1.56)	0.519
Smoking	1.92 (0.72–5.19)	1.32 (0.85–1.78)	0.148
Obesity^#^	0.92 (0.19–4.18)	0.95 (0.32–1.73)	1.000
Geographic location	1.53 (0.70–3.34)	1.23 (0.84–1.89)	0.243
BMI (25–29.9 kg/m^2^)	1.05 (0.46–2.38)	1.03 (0.64–1.52)	0.886
Tobacco consumption^#^	1.83 (0.27–15.0)	1.27 (0.45–1.85)	0.683
*Physical inactivity	4.73 (2.0–11.1)	1.95 (1.3–2.6)	<0.001

*Statistically significant variable.

*^#^Fisher’s exact test*.

## Discussion

Ischemic stroke has become a major health problem worldwide (Murray and Lopez, 1997), and therefore it is crucial to identify novel biomarkers and preventive strategies for the treatment of the disease. Brain ischemia accounts for a significant proportion of all strokes, and atherosclerosis is considered to be the major cause of most of the brain infarcts (Fuster et al., 1992; Ross, 1999). The atheromatous plaques represent a series of specific cellular and molecular responses that include lipoprotein, hematologic, and inflammatory components (Ross, 1999). Various reports have shown that inflammation may promote atherosclerosis and plaque formation by elevating serum levels of fibrinogen (Ernst and Koenig, 1997), leukocytes (Ernst et al., 1987), clotting factors (Juhan Vague et al., 1996), and cytokines (Dinerman et al., 1990) and by altering the metabolism of endothelial cells and monocyte/macrophages (Dinerman et al., 1990; Celletti et al., 2001). Viral and bacterial infections reflected in elevated levels of various acute-phase proteins (Mattila, 1989) may be partly responsible for the inflammatory processes which in turn may be associated with the occurrence of ischemic symptoms (Mattila et al., 1998). A study identified a particular protein in the sera of healthy subjects, which was initially absent in acute ischemic stroke patients and reappeared after treatment (Kashyap et al., 2006). This finding suggests that the protein may be useful as an important diagnostic marker. Moreover, several growth factors, such as basic fibroblast growth factor (bFGF), VEGF, and MCP-1 are known to play an essential role in mediating recovery in ischemic stroke patients.

The main objective of the current study was to improve our knowledge about the potential biomarkers in the sera of patients presenting with carotid plaque. Since carotid atherosclerotic lesions may develop ischemic stroke, a better understanding and identification of these biomarkers may lead to improved patient care and novel therapeutic approaches for treatment of ischemic stroke. Our study revealed no alteration in the levels of VEGF and MCP-1 after ELISA was conducted. We used various parameters such as socio-demographic variables based on certain risk factors for carotid plaque development such as advanced age, sex, hypertension, diabetes mellitus, and physical inactivity. The male/female sex ratio for stroke in India has been estimated to be 1.7:1 (Sethi, 2002). We noticed that elderly people above 60 years of age are the most affected individuals (51.4%) with men more likely to develop the disease compared to women (71.4% men). In relation to this we report a significant association of hypertension with increased risk of stroke [R 12.06 at 95% CI (4.7–31.5); *p* < 0.05]. Similarly, diabetes mellitus is identified as a putative risk factor for stroke in case-control studies. Our data showed that diabetic individuals are more vulnerable to the disease compared with the ones whose blood sugar level is normal. A significant association of diabetes with the risk of ischemic stroke identifies it to be an important risk factor in North Indian population, like previous studies [OR 4.68 at 95% CI (1.50–15.5); *p* < 0.05]. It is interesting to report there is a conflicting finding published as Dubbo study which did not report diabetes as a risk factor for stroke (Simons et al., 1998). Several lines of evidence suggest a link between physical inactivity and ischemic heart disease (Batty, 2002). Our data is consistent with the previous reports suggesting physical inactivity to be significantly associated with stroke risk (Paffenbarger and Wing, 1967; Wannamethee and Shaper, 1992; Hu et al., 2000) [OR 4.73 at 95% CI (2.0–11.1); *p* < 0.05]. This finding is consistent with the previous reports (Lanska, 1993). On the other hand no significant association of obesity, smoking, dyslipidemia, alcohol, and tobacco consumption with the stroke risk was found. The result is consistent with the Dubbo study that revealed alcohol intake and smoking as a non-determinant of stroke and indirectly supports of previous studies in which high alcohol consumption has been associated to stroke risk in comparison to moderate consumption. (Gill et al., 1991; Jamrozik et al., 1994; Caicoya et al., 1999; Malarcher et al., 2001). We also did not find any significant linkage of dyslipidemia with the risk of stroke in this report. This may be due to the fact that most of the stroke patients were being followed up and were put on medications. The current finding reveals that the prevalence of stroke was greater among stroke patients residing in urban places (Brown et al., 1996; Sacco et al., 1998; Fang, 2012) than the ones from rural areas but the difference was not statistically significant. The causes to this observation have previously been pointed out to result from less active lifestyle of urban residents than their rural counterparts (Banerjee and Das, 2006; Joshi et al., 2006). Therefore, urbanization might play a crucial role in the pathogenesis of the disease.

This finding is not in agreement with those of many previous reports from outside India where serum VEGF level was found to be upregulated in patients of ischemic stroke. The probable reason for unaltered expression of VEGF in systemic blood flow is enigmatic. We ascribe this unusual finding to limited release of this growth factor localized in and around the damaged tissue than being secreted in circulating blood stream or partly a result of activated negative feedback system (with a speculation that increased VEGF expression in ischemic penumbra may produce a molecular mediator that may turn on an inhibitory feedback mechanism). Expectedly, MCP-1 being induced by the upregulation of VEGF, its level was also found to be equally unaltered (Marumo et al., 1999). Since Cooper et al. (1999) observed an elevated expression of VEGF in experimental diabetes many studies have estimated the VEGF levels in different diseases (Slevin et al., 2000; Andrew et al., 2002; Blann et al., 2002). Also an enhanced expression of MCP-1 in patients with ischemic stroke and myocardial infarction have been observed in some studies (Arakelyan et al., 2005). It has been suggested that lifestyle of an individual has great impact on risk factors associated with stroke (Welin et al., 1987). Individuals with hypertension and diabetes have twice to sixfold chances of having stroke as per published reports (Kannel and McGee, 1979; Wolf et al., 1992; Burchfiel et al., 1994). Similarly, the results of Framingham Heart Study revealed the effect of diabetes on cerebral and peripheral arteries (Benjamin et al., 1998). Individual studies in India have estimated the prevalence rate of the ischemic stroke from 21/100,000 for individuals in 20–40 years of age group to 625/100,000 above 60 years age and 27–34/100,000 in the 35–44 age group to 822–1116/100,000 above 75 years age (Bansal et al., 1973; Razdan et al., 1989; Dhamija et al., 2000; Dalal, 2008; Sridharan, 2009). Moderate physical exercise is reported to reduce the depression rate and prevents the occurrence of heart disease and stroke by lowering the blood pressure and raising the level of HDL cholesterol in blood (Shephard, 1997; Thompson et al., 2003) which are in support to a study done in Italian population which stated that moderate amount of exercise may help in reducing the risk of stroke (Menotti and Seccareccia, 1985). The current data shows that people residing at mountain region have low rates of stroke as compared to the ones residing at plain region. Another modifiable factor, cigarette smoking, has been reported to be associated with all types of stroke including ischemic stroke, intra-cerebral, and subarachnoid hemorrhage (Shinton and Beevers, 1989; Juvela et al., 1993; Kurth et al., 2003). In addition, risk of having stroke declines when smoking is discontinued (Feigenbaum, 1993). In view of these contradictory reports, alcohol consumption, dyslipidemia, and smoking as a stroke predictor remain controversial pending population-based prospective studies.

The present study had some limitations like risk factors in other subtypes of stroke, socio-demographic parameters such as depression or stress were not assessed as well as no mechanistic information regarding stroke prevention and rehabilitation was provided. Still there is a great promise in the search for serum biomarkers to help in the prognosis of atherosclerotic disease but many theoretical and practical challenges stand in the way. Further performing studies with a large patient cohort focusing on risk factors of stroke are however, necessary to examine additional biomarkers including VEGF and MCP-1 with reduced sample time after stroke onset to corroborate this preliminary data.

## Conflict of Interest Statement

The authors declare that the research was conducted in the absence of any commercial or financial relationships that could be construed as a potential conflict of interest.

## Author Note

We confirm that we have read the Journal’s position on issues involved in ethical publication and affirm that this report is consistent with those guidelines.
